# Prevalence of Dyslipidemia in Patients with Acute Coronary Syndrome Admitted at Tertiary Care Hospital in Nepal: A Descriptive Cross-sectional Study

**DOI:** 10.31729/jnma.4765

**Published:** 2020-04-30

**Authors:** Sahadeb Prasad Dhungana, Arun Kumar Mahato, Rinku Ghimire, Rupesh Kumar Shreewastav

**Affiliations:** 1 Department of Internal Medicine and cardiology unit, Nobel Medical College Teaching Hospital, Biratnagar, Nepal; 2 Department of Internal Medicine, Nobel Medical College Teaching Hospital, Biratnagar, Nepal; 3 Department of Pharmacology, Nobel Medical College Teaching Hospital, Biratnagar, Nepal; 4 Department of Biochemistry, Nobel Medical College Teaching Hospital, Biratnagar, Nepal

**Keywords:** *acute coronary syndrome*, *dyslipidemia*, *prevalence*

## Abstract

**Introduction:**

Dyslipidemia is one of the major risk factors for acute coronary syndrome. Dyslipidemia with an increase in total cholesterol, low-density lipoprotein cholesterol, triglycerides and decrease in high-density lipoprotein cholesterol is one of the major risk factors for the acute coronary syndrome and alone account for more than 50% of population attributable risk. This study was conducted to find out the prevalence of dyslipidemia.

**Methods:**

This descriptive cross-sectional study was conducted in 105 patients admitted at the tertiary care center with a diagnosis of acute coronary syndrome from July 2018 to March 2019 after approval from the institutional review committee (Ref no. 205/2018). Fasting serum lipid profile was obtained within 24 hours of hospitalization with the convenient sampling method. Data were analyzed with the help of the Statistical Package for Social Sciences version 20. Point estimation at 95% Confidence interval was calculated along with frequency and proportion for binary data.

**Results:**

Out of 105 people, dyslipidemia was present in 51 (48.6%). The mean age of the participants was 59.19±12.69 years. The majority 81 (77.1%) were male. The mean total cholesterol, triglycerides, low-density lipoprotein cholesterol, and high-density lipoprotein cholesterol were 183.43±35.9 mg/dl, 140.59±46.83 mg/dl, 109.9±26.38 mg/dl and 41.17±4.78 mg/dl respectively. High total cholesterol and triglyceride were found in 34 (32.4%) each, low high-density lipoprotein in 31 (29.5%) and high low-density lipoprotein in 22 (21%). It was present in 8 (42.1%) underweight, 24 (40.7%) normal weight and 19 (70.4%) overweight patients. It was present in 20 (60.6%) diabetics, 34 (48.6%) hypertensives, 5 (45.5%) smokers and 2 (33.3%) with a family history of coronary artery disease.

**Conclusions:**

Dyslipidemia is a significant risk factor in patients with acute coronary syndrome and commonly associated with other risk factors. Careful attention to its management may help to reduce further events.

## INTRODUCTION

Dyslipidemia with an increase in total cholesterol (TC), low-density lipoprotein cholesterol (LDL-C), triglycerides (TG) and decrease in high-density lipoprotein cholesterol (HDL-C) is one of the major risk factors for the acute coronary syndrome (ACS)^1^ and alone account for more than 50% of population attributable risk.^2^ Untreated dyslipidemia is the strongest predictor of in-hospital death.^3^

There is an agreement that the measurement of TC within the first 24 hours of myocardial infarction (MI) reflects its habitual value.^4,5^ Therefore, the guidelines suggest that the lipid profile be measured within that time interval.^6^ Dyslipidemia is an independent and modifiable risk factor that is common in our population. It remains unrecognized until detected during the first presentation with ACS.

So, this study aimed to find out the prevalence of dyslipidemia within 24 hours of admission with ACS and thereby help to the early classification of dyslipidemia and select the appropriate therapy.

## METHODS

This is a descriptive cross-sectional study, which was conducted on 105 admitted patients diagnosed with ACS at Nobel Medical College Teaching Hospital from July 2018 to March 2019 after getting the approval from the Institutional review committee (Ref no. 205/2018). All the participants had signed the consent for the study. History and clinical examination were performed as per the pre-structured proforma. All cases above the age of 20 years with a diagnosis of ACS were enrolled by a convenience sampling method. Patients with stable coronary artery disease were excluded. The sample size of 105 was calculated based on the 62% prevalence of dyslipidemia in patients with ACS in the study conducted at another tertiary care hospital in Nepal^7^ by using formula

$$\begin{array}{ll} \text{n} & \text{= }{\text{Z}}^{\text{2}}\times \text{p }\times \text{q}/{\text{e}}^{\text{2}}\\ & \text{= }{(\text{1.96)}}^{\text{2}}\times \text{62}\times \text{38}/{\text{(}9.\text{3)}}^{\text{2}}=3.84\times 2356/\text{86.49}\\ & \text{= }104.6 \end{array}$$

Where,

n = required sample size
p = prevalence of condition (62%)^7^
q = 1-p
e = margin of error (15% of 62 = 9.3)

Where, the 5% level of significance (Z= 1.96) and 15% permissible relative error, hence, the sample size was 105 cases.

An electrocardiogram (ECG) at presentation was performed in all the patients and the cases were categorized as unstable angina (USA), non-ST elevation MI (NSTEMI) and ST-elevation MI (STEMI) based on the history, ECG changes and cardiac markers.^8^ Fasting serum lipid profile was obtained within 24 hours of hospitalization. Lipid assay was done with Enzymatic Colorimetric Test for TC and TG with lipid Clearing Factor. LDL-C was determined by direct method and HDL-C by precipitation method.

Dyslipidemia was defined based on NCEP ATP III criteria^9^ i.e. any of the following fasting lipid profile values obtained within 24 hours of the event: TC ≥ 200 mg/dl, TG ≥ 150 mg/dl, LDL ≥ 130 mg/dl, and HDL ≤ 40 mg/dl or patient already on medication for dyslipidemia. Dyslipidemia was evaluated with age, sex, and other risk factors.

The data were collected and entered in MS-Excel 2007 and analyzed using the Statistical Package for Social Sciences (SPSS) version 20 software.

## RESULTS

Out of 105 patients, 81 (77.1%) were male and 24 (22.9%) were female. The mean age was 59.19±12.69 years. Sixty-four (61%) participants had STEMI, 27 (25.7%) NSTEMI and 14 (13.3%) USA. Hypertension was present in 35 (33.3%) and was the most frequently observed risk factor followed by diabetes 33 (31.4%). Twenty-seven (25.7%) patients were overweight or obese, 11 (10.5%) patients were smokers, 5.7% had a family history of CAD and 4.8% had a history of significant alcohol consumption (Table 1).

**Table 1:** Prevalence of risk factors for the acute coronary syndrome.

Risk factors	Total n (%)	Male n (%)	Female n (%)
Hypertension)	35 (33.3)	25 (30.9)	10 (41.7)
Diabetes mellitus	33 (31.4)	25 (30.9)	8 (33.3)
Overweight or obese	27 (25.7)	22 (27.2)	5 (20.8)
Smoker	11 (10.5)	6 (7.4)	5 (20.5)
Family history of CAD	6 (5.7)	5 (6.2)	1 (4.2)
Alcohol user 6 (5.7)	5 (4.8)	4 (4.9)	1 (4.2)
Total	105 (100)	81 (77.1)	24 (22.9)

CAD = Coronary Artery Disease

The mean TC, TG, LDL-C and HDL-C among the participants was 183.43±35.91 mg/dl, 140.59±46.83 mg/dl, 109.90 ± 26.38 mg/dl and 41.17 ± 4.78 mg/dl respectively. Similarly, it was calculated for both males and females separately (Table 2).

**Table 2:** The pattern of lipid profile in the study population.

Lipid profile	Total (mean ± SD)	Male (mean ± SD)	Female (mean ± SD)
TC (mg/dl)	183.43 ± 35.91	82.14 ± 36.41	187.92 ± 34.50
TG (mg/dl)	140.59 ± 46.82	142.17 ± 50.37	135.25 ± 32.39
LDL-C (mg/ dl)	109.90 ± 26.37	107.10 ± 24.26	119.38 ± 31.24
HDL-C (mg/dl)	41.17 ± 4.77	41.48 ± 4.49	40.12 ± 5.60

*TC=Total Cholesterol, TG = Triglycerides, LDL-C = Low Density Lipoprotein Cholesterol, HDL-C= High Density Lipoprotein Cholesterol

**Figure 1.**
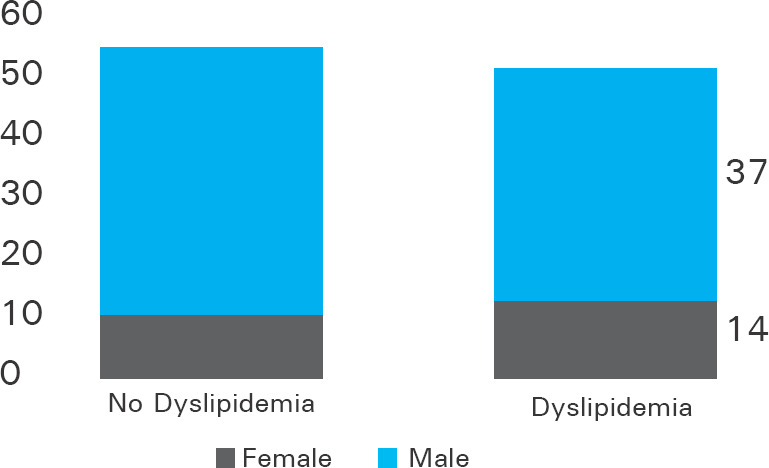
Bar diagram showing dyslipidemia according to sex.

Dyslipidemia was present in 51 (48.6%) patients. Prevalence of dyslipidemia was 14 (58.3%) in female and 37 (45.7%) in male (Figure 1).

It was more frequent in age group ≤ 45 years (58.8%) than in age group > 45 years (46.6%). Dyslipidemia was present in 9 (64.3%) patients with USA, 13 (48.1%) with NSTEMI and 29 (45.3%) with STEMI. Dyslipidemia was present in 8 (42.1%) of underweight (BMI <18.5 kg/m2), 24 (40.7%) in normal weight (BMI 18.5 - 24.9 kg/m2) and 19 (70.4%) in overweight and obese patients (BMI ≥25 kg/m^2^). Dyslipidemia was present in 34 (48.6%) of non-hypertensive and hypertensive patients both, 20 (60.6%) of diabetic and 31 (43.1%) of non-diabetic patients. Two (33.3%) of patients with a family history of CAD had dyslipidemia and 49 (49.5%) of patients without a family history of CAD had dyslipidemia. Dyslipidemia was present in 46 (48.9%) of non-smoker and 5 (45.5%) of smokers whereas it was present in 4 (80.0%) of alcohol consumer and 47 (47.0%) of patients who did not consume alcohol. Among the diabetic participants, 20 (60.6%) was found to have dyslipidemia and similarly patients with normal BMI has less percentage of dyslipidemia (Table 3).

**Table 3:** Distribution of dyslipidemia according to risk factors.

Risk factors	No dyslipidemia	Dyslipidemia
No family history of CAD	50 (50.5)	49 (49.5)
Family history of CAD	4 (66.7)	2 (33.3)
Non-hypertensive	36 (51.4)	34 (48.6)
Hypertensive	18 (51.4)	17 (48.6)
Non diabetic	41 (56.9)	31 (43.1)
Diabetic	13 (39.4)	20 (60.6)
Non smoker	48 (51.1)	46 (48.9)
Smoker	6 (54.5)	5 (45.5)
No history of alcohol use	53 (53.0)	47 (47.0)
Significant alcohol consumer	1 (20.0)	4 (80.0)
Underweight	11(57.9)	8 (42.1)
Normal BMI	35 (59.3)	24 (40.7)
Overweight and obese	8 (29.6)	19 (70.4)

* CAD=coronary artery disease, BMI=body mass index

The high serum level of TC was found in 34 (32.4%), high serum level of TGs in 34 (32.4%), high serum level of LDL-C in 22 (21.0%) and low serum level of HDL-C in 31 (29.5%) (Table 4).

**Table 4:** Distribution of dyslipidemia according to TC, TG, LDL-C, and HDL-C.

Lipid profile	Categories	Dyslipidemia n (%)	Total n (%) TC in mg/dl
TC in mg/dl	<200	17 (33.3)	71 (67.6)
	≥200	34 (66.7)	34 (32.4)
TG in mg/dl	<150	17 (33.3)	71 (67.6)
	≥150	34 (66.7)	34 (32.4)
LDL-C in mg/dl	<130	29 (56.9)	83 (79.0)
	≥130	22 (43.1)	22 (21.0)
HDL-C in mg/dl	>40	20 (39.2)	74 (70.5)
	≤40	31 (60.8)	31 (29.5)

Abbreviations: TC; total cholesterol, TG; triglycerides, LDL-C; low-density lipoproteins, HDL-C; high-density lipoproteins

The prevalence of high TC, high LDL-C, and low HDL-C were non-significantly high in females and the prevalence of high TG was non-significantly more in males.

## DISCUSSION

This study was conducted to determine the prevalence and pattern of dyslipidemia in patients with ACS from a tertiary care hospital in the eastern part of Nepal. In this study, the lipid profile analysis demonstrated the presence of some type of dyslipidemia in 48.6% of all patients with no significant difference in male and female, with high TC and TG levels being the highest prevalence. Likewise, a study done at the National Heart Centre in Nepal^10^ found the prevalence of dyslipidemia in 45.5% of patients with no significant difference between genders.

In this study, the majority of the patients had one or more conventional risk factors for CAD. The common risk factors were dyslipidemia, diabetes, and hypertension which are similar to other studies^11,12^ which demonstrated a high prevalence of one or more major risk factors for CAD and ACS. Majority of patients were male (77.1%) suggesting that male gender as one of the risk factors for ACS as seen in previous study.^13^

Dyslipidemia is an independent major risk factor for CAD. Studies have reported a higher prevalence of dyslipidemia among Asians compared to the western population.^14^ A combination of low HDL-C and high TG referred to as atherogenic dyslipidemia, have been implicated as important predictors of CAD.^15,16^

In this study, the most commonly observed lipid abnormality was high TC and TG followed by low HDL-C and high LDL-C. Previous studies on CAD patients also found similar results regarding the presence of high levels of TC, LDL-C, and TG and low levels of HDL-C.^17,18^ The lower prevalence of high LDL-C as compared to other fractions is consistent with previous study^14^ which showed a higher prevalence of low LDL levels among Asians. Our study showed low HDL-C levels in a high percentage of patients (60.8%) as shown in the previous study which revealed that South Asians had lower HDL-C levels than rest of the other population.^14^

These findings may have clinical importance since the prior study in patients with CAD who had a low HDL-C was associated with increased risk for death and MI, even among patients achieved LDL <70 mg/dl.^19^

We assessed the effect of other major risk factors of CAD on the prevalence of dyslipidemia. We did not find a significant difference in the prevalence of dyslipidemia among those with or without major risk factors except for obese or overweight patients who had a higher burden of dyslipidemia similar to a previous study showing that BMI and central obesity is associated with atherogenic dyslipidemia.^20^

This study provides the pattern of lipid abnormalities early after ACS among the Nepalese population and guides to the early classification of dyslipidemia and the selection of lipid-lowering therapy. This is a hospital-based study in a small number of patients. Lipid profile was obtained within the first 24 hours of the event and baseline values were not available for comparison. We did not look at the apolipoprotein measures (Apo B and Apo AI) which are strongly associated with the risk of MI in South Asians.

## CONCLUSIONS

There is a lower prevalence of high LDL-C and a high prevalence of lower HDL-C levels among the Nepalese population. Although Nepalese patients are likely to benefit from lowering LDL-C, the threshold for treatment and targets seem to be lower. These thresholds and targets need to be determined by large studies and by our guidelines. Given the lower levels of HDL-C, approaches to increase the HDL-C may be helpful in our population.

## Conflict of Interest

**None.**
